# Who misses MCI diagnosis before dementia?

**DOI:** 10.1002/alz70861_108609

**Published:** 2025-12-23

**Authors:** Chao‐Yi Wu, Yingnan He, Hiroko H Dodge, Sudeshna Das

**Affiliations:** ^1^ Massachusetts General Hospital, Harvard Medical School, Boston, MA USA

## Abstract

**Background:**

Mild cognitive impairment (MCI), an intermediate stage between normal cognition and dementia, represents a key opportunity for early intervention, especially with emerging anti‐amyloid therapies. However, many individuals delay seeking medical care until symptoms exacerbate, resulting in a dementia diagnosis without a prior MCI diagnosis – thus missing a critical window for intervention. This study examined the risk factors associated with the absence of an MCI diagnosis prior to a dementia diagnosis.

**Method:**

Electronic health record (EHR) data were extracted from the Massachusetts General Brigham (MGB) Cloud Enterprise Data Warehouse. Individuals aged ≥ 21 years who presented with normal cognition at their first hospital encounter and were subsequently diagnosed with dementia were included. Diagnoses were identified using ICD‐10‐CM codes for normal cognition, MCI (G31.84), and various types of dementia (e.g., G30.9, G31.0, F01.5). Covariates included demographics, state‐level rank of the Area Deprivation Index (ADI), Charlson comorbidity index (CCI), and indicators of routine and preventative care (vaccinations; annual wellness visits; routine screenings). Logistic regression models were applied to identify factors associated with missed MCI diagnosis, controlling for age and duration in the EHR.

**Result:**

Among 32,430 individuals (mean age at first hospital encounter 76.6±10.9 years; 59.1% female; 4.2% African American; 5.0% Hispanic), only 16.0% received an MCI diagnosis before their dementia diagnosis. African American and Hispanic patients had 31% and 44% lower odds (OR=0.69, *p* <.001; OR=0.56, *p* <.001), respectively, of receiving an MCI diagnosis before dementia in comparison to White non‐Hispanic patients. Individuals with at least a high school education (OR=1.37, *p* <.001) and those who were married (OR=1.51, *p* <.001) were more likely to be diagnosed with MCI before dementia. Greater socioeconomic adversity and a higher comorbidity burden were associated with lower odds of receiving an MCI diagnosis (OR=0.92, *p* <.001; OR=0.98, *p* =0.04). Routine care utilization, including vaccinations and annual wellness visits, was linked to increased likelihood of MCI detection prior to dementia diagnosis (OR=1.48‐1.67, *p* <.001).

**Conclusion:**

Sociodemographic and routine healthcare utilization factors strongly influence the likelihood of receiving an MCI diagnosis prior to dementia within a healthcare system. These findings highlight strategies to improve early detection of cognitive impairment.

